# The relationships between golf and health: a scoping review

**DOI:** 10.1136/bjsports-2016-096625

**Published:** 2016-10-03

**Authors:** A D Murray, L Daines, D Archibald, R A Hawkes, C Schiphorst, P Kelly, L Grant, N Mutrie

**Affiliations:** 1 Physical Activity for Health Research Centre, University of Edinburgh, Edinburgh, UK; 2 Sport and Exercise, University of Edinburgh, Edinburgh, UK; 3 Usher Institute of Population Health Sciences and Informatics, University of Edinburgh, Edinburgh, UK; 4 Scottish Collaboration for Public Health Research and Policy, University of Edinburgh, Edinburgh, UK; 5 European Tour Performance Institute, Virginia Water, UK; 6 Sports and Exercise Medicine, University College London, London, UK; 7 Global Health Academy, University of Edinburgh, Edinburgh, UK

**Keywords:** Golf, Health, Exercise, Evidence based review, Sport

## Abstract

**Objective:**

To assess the relationships between golf and health.

**Design:**

Scoping review.

**Data sources:**

Published and unpublished reports of any age or language, identified by searching electronic databases, platforms, reference lists, websites and from consulting experts.

**Review methods:**

A 3-step search strategy identified relevant published primary and secondary studies as well as grey literature. Identified studies were screened for final inclusion. Data were extracted using a standardised tool, to form (1) a descriptive analysis and (2) a thematic summary.

**Results and discussion:**

4944 records were identified with an initial search. 301 studies met criteria for the scoping review. Golf can provide moderate intensity physical activity and is associated with physical health benefits that include improved cardiovascular, respiratory and metabolic profiles, and improved wellness. There is limited evidence related to golf and mental health. The incidence of golfing injury is moderate, with back injuries the most frequent. Accidental head injuries are rare, but can have serious consequences.

**Conclusions:**

Practitioners and policymakers can be encouraged to support more people to play golf, due to associated improved physical health and mental well-being, and a potential contribution to increased life expectancy. Injuries and illnesses associated with golf have been identified, and risk reduction strategies are warranted. Further research priorities include systematic reviews to further explore the cause and effect nature of the relationships described. Research characterising golf's contribution to muscular strengthening, balance and falls prevention as well as further assessing the associations and effects between golf and mental health are also indicated.

## Introduction

The objective of this scoping review is to map the literature on golf and health and to examine the relationships and effects of golf on physical and mental health.

Golf is a sport usually played on a large open-air course, in which a ball is struck with a club, with the aim of taking the lowest number of shots possible to get the ball into a series of holes in the ground. Golf is played by around 55 million people^1^ in 206 countries worldwide^2^ representing 1/127 of the global population. This global reach, and appeal to persons of all ages and abilities has seen golf reintroduced in 2016 to the Olympic Games, with efforts ongoing to secure Paralympic status for disability golf. Further information about golf is shown in online supplementary appendix 1.

10.1136/bjsports-2016-096625.supp1 Supplementary appendix

Health is influenced by a range of individual behaviours and characteristics, and the physical, social and economic environment that people are subject to.^3^ There is compelling evidence that regular physical activity has longevity, physical and mental health benefits for people of all ages, genders, geographical and socioeconomic backgrounds, and can deliver economic benefits for communities, as well as national and international policymakers.^4–6^

Golf has potential to provide physical activity, and thus health and social benefits to persons of all ages. Golf is particularly popular among middle-aged and older adults, who are generally less active than younger adults.^7^ ^8^ To date the review evidence on this topic is limited. Previous reviews,^9^ ^10^ including a systematic review,^11^ have been undertaken to consider the relationships between golf and health with many of these focusing on the subject of golf-related injuries, while a further review of undocumented methodology^12^ focused on health benefits only. A recent systematic review of health benefits related to sport suggested that evidence was conclusive only for football (soccer) and running, noting further evaluation and research looking at other sports, including golf, was required.^13^ A clear need exists to comprehensively review the relationships between golf and health. We therefore undertook a scoping review that maps available evidence, in order to identify the existing gaps in evidence and document impacts of golf on health where these data were available.

## Methods

We adopted the established five-stage scoping review process proposed by Arksey and O'Malley, incorporating adaptions from Levac *et al*, and the Joanna Briggs Institute^14–16^ as per our published protocol.^17^ The following summarises our approach to each stage.

- **Stage 1: Identify the research question**

Considering the populations, concepts and contexts of interest enabled a broad research question to be formulated:

What is known about the relationships and effects of golf on physical and mental health?

- **Stage 2: Identifying relevant studies**

The following explicit inclusion and exclusion criteria were developed through researcher discussion and expert consultation:

*Inclusion criteria:*

- Research articles not limited by geographical location, language or setting.
- All age groups and both sexes of participants.
- Research that considers the general population, as well as specific population groups (with a specific physical or mental illness or condition).
- All forms of golf (including but not limited to 18 holes, 9 holes, driving range, spectating).
- Any physical and/or mental health condition.
- Sources of information, including primary research studies, reviews, systematic reviews, scoping reviews, meta-analyses, guidelines, as well as grey literature to include unpublished and ongoing trials, annual reports, dissertations and conference proceedings.

*Exclusion criteria:*

- Opinion pieces/opinions, magazine and newspaper articles, case reports, papers with no data.
- Health and safety/occupational issues not related to playing or watching golf.
- Studies focusing on biomechanics, or improved performance in golf.

### Search strategies and databases

*Step 1:* An initial limited search

An initial limited search (September 2015) of SPORTDiscus and Google Advanced Search for review articles and ProQuest for dissertations was conducted as detailed in the published protocol.^17^

*Step 2*: Identify key words and index terms

The title, abstract and index terms used to describe the articles identified in step 1 were analysed. The research team identified golf as the only primary research term. For the health-focused databases, namely MEDLINE and PsycINFO, ‘golf’ was used as the only search term to maximise inclusivity. Secondary search terms included a broader set of keywords for SPORTDiscus, Web of Science and Google Scholar. Boolean terms AND and OR were used to extract relevant studies. All relevant articles from SPORTDiscus and Web of Science were reviewed, with the same search strategy applying to Google Scholar. A pragmatic decision to review only the Google Scholar articles with these terms in the title was taken following consultation with a research librarian.

A similar strategy was applied to the grey literature. The same search terms used for SPORTDiscus, Web of Science and Google Scholar were applied to search for theses in the ProQuest database. ‘Golf’ as the only search term was used for the WHO International Clinical Trials Registry Platform. The advanced search function on Google was used to look for relevant reports and articles from the World Golf Foundation, the Royal and Ancient, the *British Journal of Sports Medicine*, The American College of Sports Medicine and the Faculty of Sports and Exercise Medicine while representatives of these organisations were contacted for further information.

*Step 3*: Further searching of references and citations

A search was conducted of the reference list of the most relevant identified articles while authors of relevant primary comprehensive, scoping or systematic reviews were contacted for further information.

The complete final search strategy is shown in online supplementary appendix 2.

- **Stage 3: Study selection**

10.1136/bjsports-2016-096625.supp2 Supplementary appendix

Relevant titles and abstracts were evaluated against the eligibility criteria by one reviewer (ADM). A second reviewer (LD) completed the same process on a random sample of 10% of titles and abstracts, with concordance >97% regarding inclusion/exclusion decision. Where a consensus was not reached, the study proceeded to full-text review.

Scoping reviews are typically iterative, as reviewers become increasingly familiar with the research and evidence.^14^ We wished to focus on the relationships and effects of golf on physical and mental health. To enhance this focus, ‘studies focusing on biomechanics, or improved performance in golf’ was added to the existing exclusion criteria stated in the scoping review protocol.^17^

Full-text articles meeting the inclusion criteria were sourced. Translations by University staff and associates who were fluent speakers of Chinese, French, German, Italian, Japanese, Korean, Spanish and Thai to English were undertaken. Despite searching the University of Edinburgh library databases, using interlibrary loans and contacting authors, 3^18–20^ of 365 papers could not be found and were excluded.

- **Stage 4: Charting the data**

### Extracting the results

Charting tables to record and assimilate extracted data from included studies were developed. *A priori* categories were charted as were emergent themes. Three reviewers (ADM, LD and EJ) undertook data extraction duties. A sample data extraction form is shown in online supplementary appendix 3. ADM extracted data from 90% of included studies and LD/EJ extracted data from 10% of studies. LD/EJ checked 10% of ADM's data extractions for accuracy and vice versa. Any discrepancies were discussed at group meetings. Concordance was >97% regarding inclusion/exclusion.

Data extraction categories

- Author(s).
- Year of publication.
- Origin (where the study was published/conducted).
- Aims/purpose.
- Study population and sample size (if applicable).
- Methodology/methods.
- Intervention type, comparator, details of these.
- Duration of the intervention.
- Outcomes and details of these (eg, how measured).
- Key findings that relate to the scoping review research questions.

10.1136/bjsports-2016-096625.supp3 Supplementary appendix

**Stage 5: Collating, summarising and reporting the results**

Methods employed in the protocol^17^ enabled us to collate existing knowledge on this broad topic and summarise and report as

1. A descriptive analysis, mapping the data, showing distribution of studies by period of publication, country of origin, study method and theme/focus.
2. A thematic summary, describing how identified research relates to the research question and aims, and the main findings from these organised by theme.

In this study, we aim to:

- Map the evidence and key concepts available for golf and health.
- Summarise and share existing research findings in a useful way for policymakers, practitioners and other relevant stakeholders.
- Identify research gaps in the existing literature on golf and health.

## Results and discussion

### Descriptive analysis

**Figure 1:**
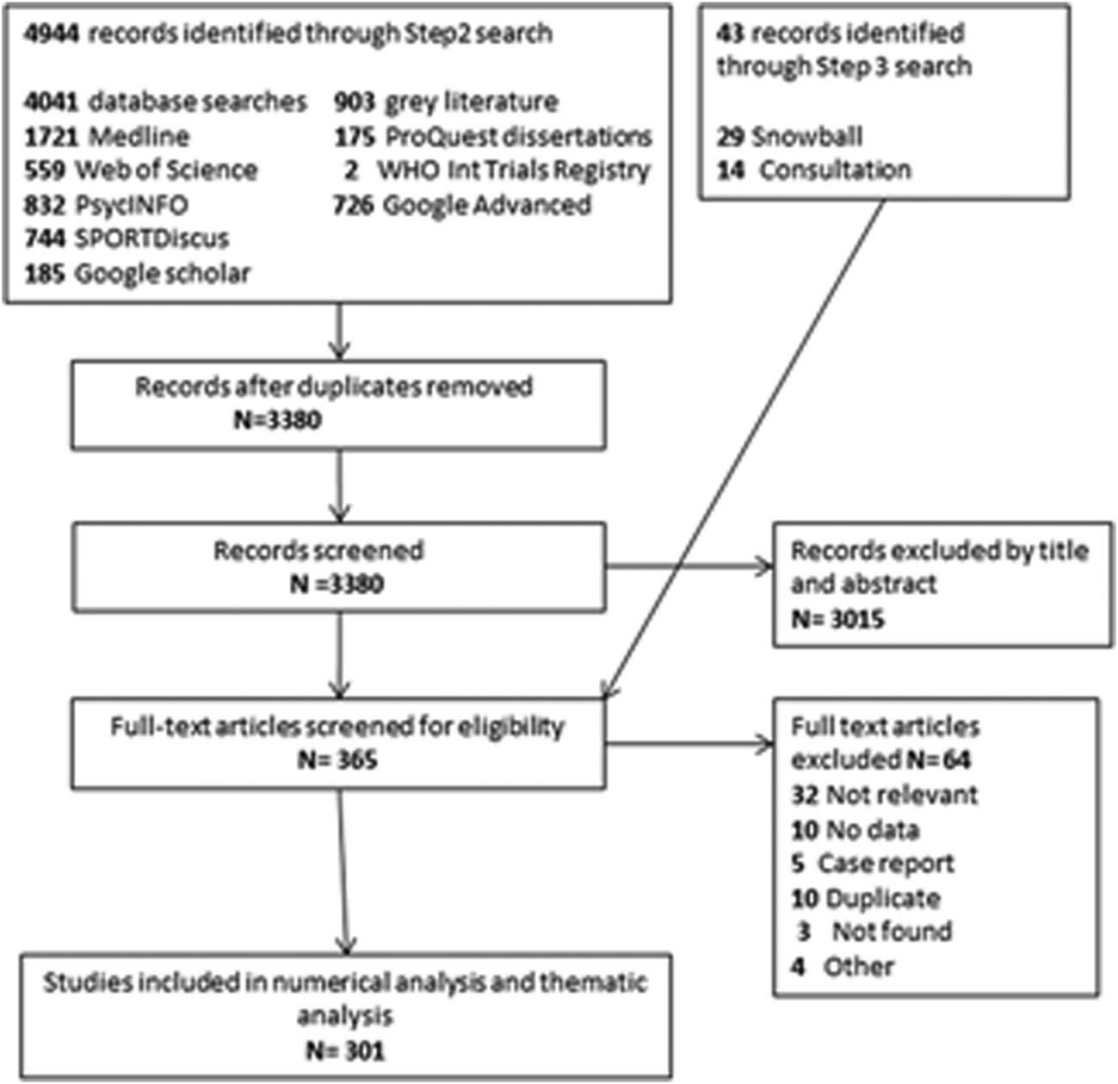
Scoping review flow chart.

A review flow diagram (see figure 1) details the results from the search, and study selection processes.

Our initial search identified 4944 studies. Of these, 4041 were identified searching databases/search platforms, and 903 from grey literature. After duplicates were excluded, 3380 records remained. A further 43 eligible studies were identified by snowballing or via expert consultation during the step 3 search.

In total, 362 articles underwent full-text screening, 3015 records being excluded after abstract screening with a further 3 articles excluded as full text was unavailable.

Overall, the scoping review identified 301 eligible studies relevant to the aims and research question ‘What is known about the relationships and effects of golf on physical and mental health?’ and these are included in the analysis.

#### Included studies by year of publication

**Figure 2:**
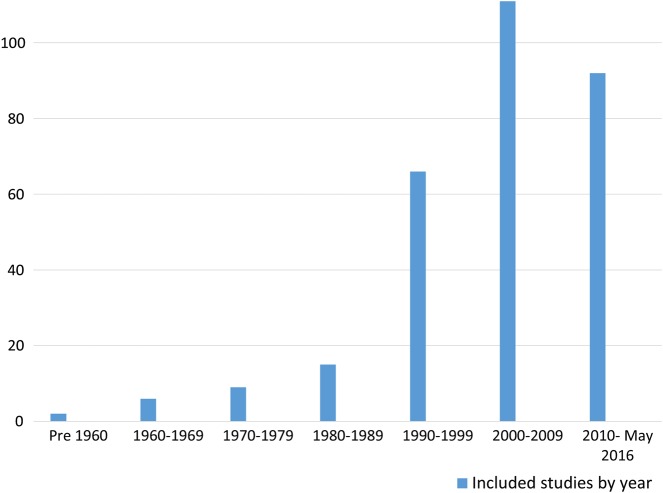
Included studies by year of publication.

In keeping with wider bibliometric trends in sport and health research, figure 2 highlights a substantial chronological increase in the number of papers relating to golf and health, with an associated increase in the range of study designs and research questions.

#### Geography of included studies

Research studies were identified from 24 countries and in 9 languages. Table 1 demonstrates the percentage of included studies per country. The majority (53.8%) of included studies were from the USA, where almost half of the world's golfers live.^2^ Studies from North America (57.1%), Europe (22.3%) and Oceania (10.0%) are relatively well represented, as they are generally for research publications on physical activity.^21^ There were fewer included studies per golfing facility (eg, golf course, driving range and practice facilities) from Asia (10.0%) and Africa (0.3%), and none included from South America.

**Table 1:** Geography of included studies

Country	No. of studies	Percentage of studies
USA	162	53.8
UK	38	12.6
Australia	27	9.0
Japan	12	4.0
Canada	10	3.3
South Korea	10	3.3
Germany	8	2.7
China	4	1.3
Sweden, Norway, New Zealand, Switzerland, Spain, France	3 each	1.0 each
Finland, Austria, Thailand	2 each	0.7 each
India, Singapore, the Netherlands, South Africa, Italy	1 each	0.3 each
All	301	99.9

### Type of study

#### Study design

**Figure 3:**
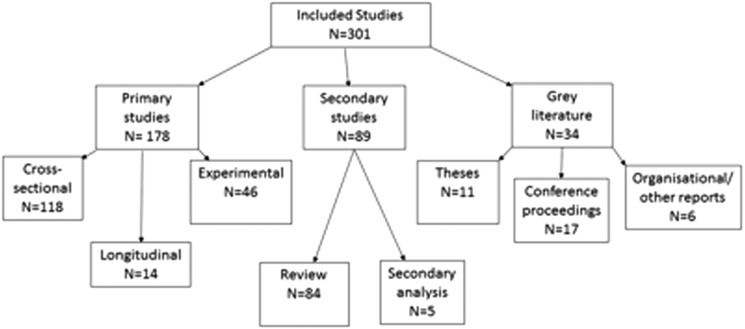
Taxonomy of research designs for included literature.

The studies varied considerably in terms of study design and primary focus. No formal quality assessment of included studies was performed as scoping reviews are intended to provide a map of what evidence has been produced as opposed to seeking only the best available evidence to answer a narrow policy or practice-related question.^15^ A taxonomy of research designs included by the scoping review is shown in figure 3.

One hundred and seventy-eight (59.1%) were primary research, while 89 (29.5%) were secondary studies and 34 (11.3%) were grey literature.

Of the primary literature, 118 (66.3%) studies had a cross-sectional design, with 14 (7.9%) longitudinal and 46 (25.8%) experimental. The majority of the experimental studies quantified golf parameters, for example, steps taken or calories burned while playing golf. Overall 16 of 301 studies conducted a primary assessment of health outcomes in relation to golf, while only 4 conducted interventions principally aiming to promote behaviour change in relation to golf and health.

The vast majority of secondary studies were reviews. Only six of these were systematic reviews. The systematic reviews each focused on a narrow aspect of the broad topic of golf and health.

The grey literature comprised 17 published conference proceedings, 11 theses and 6 organisational reports.

#### Theme of the study

The primary focus of the included studies fitted broadly into four key themes, namely

1. Physical activity and golf (N=49).
2. Golf and physical health (non-injury/accident) (N=49).
3. Golf and injury/accident (N=135).
4. Golf and mental health/wellness (N=29).

**Figure 4:**
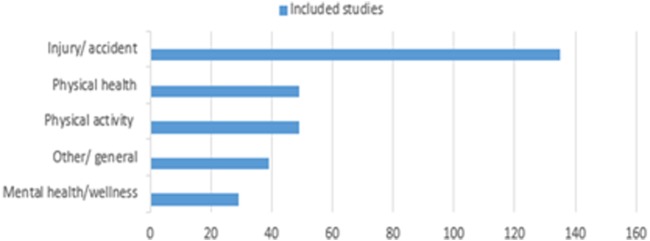
Primary focus of included studies.

These themes were formed from merging of the *a priori* categories identified. Additional studies from emergent themes were classified into a further category ‘other and general’ (N=39) to include studies of golf participation, implications for policy, legal implications or studies that focus evenly on more than one of these areas. Articles focusing on injuries and accidents relating to golf were the most frequent, comprising nearly 44.9% of included studies despite the exclusion of articles with a biomechanical/performance focus. Figure 4 shows the primary focus of included studies.

## Thematic summary

### Key concepts and evidence available

#### Participation

Golf is a sport played by 55 million people in 206 countries, by males and females across the life-course.^1^ ^2^ Globally, this compares to 250 million direct participants in football (soccer),^22^ 75 million tennis^23^ and 5 million rugby union players.^24^ Gaining health benefits and exercise are powerful motivators for persons to play sport, and golf in particular.^25–27^ Golfers more frequently continue to play into middle age compared with participants in sports like football and rugby.^28^ ^29^ Golf is played by people of all backgrounds, but participation is stronger in males,^8^ ^30^ higher socioeconomic groups^7^ and more affluent countries.^2^

#### Golf and physical activity

Golf can contribute to physical activity as a leisure time or recreational activity, while work and occupation yields physical activity for modest numbers of professional players and caddies.^31^

The relative contribution of golf to population physical activity increases in older adults,^27^ ^31^ a group that are typically less physically active than younger adults,^32^ but for all ages remains considerably less than recreational walking, which is highly accessible and often bears zero cost.^31^

Individual differences in energy expenditure can be large, depending on individual and golf-related factors, but golf can provide moderate intensity physical activity. Moderate intensity physical activity is recommended for children, adults and older adults for the longevity, physical and mental health effects it brings.^33–35^ Golf typically involves a mixture of exercise intensities. Golf can help persons and populations meet, and exceed minimum health and government recommendations for Moderate to Vigorous Physical Activity.^36^ ^37^

**Figure 5:**
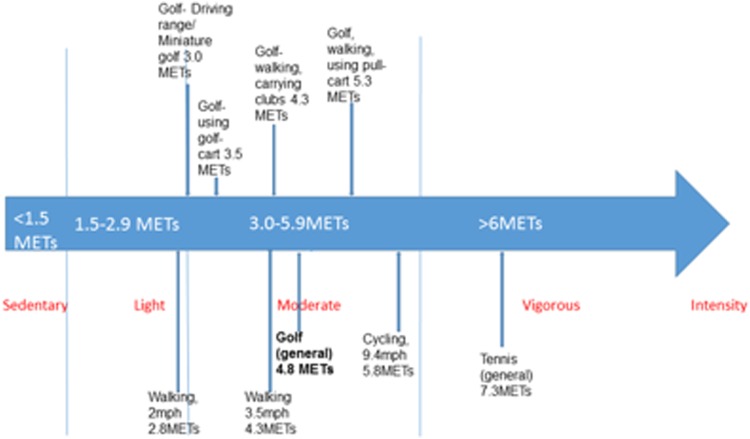
MET values attributed to different modes of golf and other physical activities. MET, Metabolic Equivalent of Task.

Studies quantifying golf by Metabolic Equivalent of Task (MET) value generally agree it offers moderate intensity aerobic activity,^36^ ^38–46^ although with a wide range of MET values quoted (2.5–8.0) some studies classify it as low intensity^47^ ^48^ or high intensity.^49^ ^50^ The mean of the range of estimates is 4.5 METs. Figure 5 shows MET values attributed to different modes of golf and, for comparison, other physical activities suitable for all ages, by the Compendium of Physical Activity.^38^

Studies assessing calorie expenditure during golf typically classify golf as a moderate intensity physical activity with energy expenditure of 3.3–8.15 kcal/min,^41^ ^42^ ^51–56^ 264–450 kcal/hour^51^ ^52^ ^56^ and a total energy expenditure of 531–2467 kcal/18 holes.^36^ ^42^ ^43^ ^47–49^ ^51–53^ ^56–59^ Golfers walking 18 holes take between 11 245 and 16 667^36^ ^49^ ^60^ ^61^ steps, walking 4–8 miles,^36^ ^48^ ^49^ ^51^ ^57^ ^60^ ^62^ while those playing and riding a golf cart accrue 6280 steps^61^ or just under 4 miles.^51^ There is poor agreement in the literature assessing intensity of golf by heart rate, with a majority classifying golf as low intensity,^47^ ^58^ ^63–65^ but others quantifying it as moderate to high intensity.^36^ ^57^ ^66^ ^67^

Intensity of physical activity playing golf is higher for those walking rather than riding a golf cart,^51^ ^58^ ^59^ ^65^ ^68^ those playing a hillier course,^55^ ^67^ older adults,^66^ heavier players,^49^ ^56^ ^69^ males^36^ and those of low baseline fitness. Intensity further varies depending if a player is swinging a club, walking or standing.^69^

Knowledge of the contribution of golf to muscle strengthening and to the balance aspects of physical activity recommendations is limited, and a priority for a review and further primary research. Studies suggest that golf may improve proprioception, balance, muscle endurance and function particularly in the elderly,^57^ ^70–74^ while in younger players, no increase in muscle mass or bone mineral density has been seen.^75^

Sedentary behaviour is characterised as ‘any waking activity characterised by an energy expenditure over 1.5 METs and a sitting or reclining posture’.^76^ Time playing golf without riding a golf cart is non-sedentary time,^36^ ^38–45^ ^47–49^ and although golfers riding a golf cart do gain some health-enhancing physical activity, golfers walking the course gain more.

Unlike most other sports, golf spectating offers the opportunity to walk around the field of play, rather than being restricted to a seat. Spectators from North America and South Korea have highlighted ‘exercise’ as a reason for attending golf events, which can attract in excess of 500 000 spectators per week.^77–79^

#### Golf and longevity

Physical inactivity is a determinant of excess mortality, killing >3 million,^5^ and perhaps in excess of 5 million people annually.^4^ The 2010 Global Burden of Disease study highlights that physical inactivity is one of the top five causes of death in North America, Western Europe and in Australasia,^5^ three regions where golf is frequently played.^2^

The best available evidence suggests that playing golf may contribute to reduced mortality and increased life expectancy. When a Swedish study compared 300 818 golfers to non-golfers, they found a 40% lower mortality rate, although the study design and limitations meant that this could not be directly attributed to golf-related physical activity.^80^ The authors of that study speculate that this corresponds to a 5-year increase in life expectancy regardless of gender, age or socioeconomic status. This increase will also have further contributing factors, including other lifestyle factors. Playing sport several times per week is likely to benefit health more than playing one to two times per week.^81^ An association, but not causal relationship, is demonstrated between golf and life expectancy in Swedish and US studies.^80^ ^82^

#### Golf and physical health

In providing moderate intensity physical activity, it is biologically plausible that golf could be expected to have beneficial effects in the prevention and treatment of chronic diseases, including ischaemic heart disease, type 2 diabetes, stroke, and colon and breast cancer.^4^ ^83^ A review commissioned by the World Golf Foundation concluded that participating in golf can ‘yield a number of positive health and fitness effects’^12^ although methods were not stated and only health benefits were described. Frequent golfers perceive their physical health to be better than infrequent golfers.^84^

##### Cardiovascular system

Golf is associated with improvements in known risk factors for cardiovascular disease, including physical inactivity,^38^ blood lipid and insulin–glucose levels,^57^ ^66^ ^85^ body composition^57^ ^85^ and aerobic fitness,^57^ although direct evidence and longitudinal trials assessing the medium-term and long-term impact of golf on coronary heart disease or cerebrovascular disease are lacking. Golf is reported as providing suitable exercise for patients with cardiac^43^ ^86^ ^87^ and stroke rehabilitation.^88^

Golf can provide a sufficient stimulus to improve aerobic fitness, but higher intensity exercise generates significantly improved cardiovascular adaption compared to playing golf.^43^ ^57^ ^69^ The effects of a season of golf on systolic blood pressure showed no significant difference in a controlled trial,^57^ while no consistent effect has been found measuring blood pressure during golf.^64^ ^67^ ^86^

There is an increased incidence of acute cardiac events during participation in sport^89^ and golf in particular.^90–92^ Golf players with new or unstable cardiac symptoms should consult a doctor.^90^ There is contradictory and inconclusive evidence regarding the effectiveness and cost-effectiveness of automatic external defibrillators situated at golf courses.^93–96^ An extremely rare mechanism of ischaemic stroke linked to golf has been described.^97^

##### Respiratory system

Regular participation in golf may improve lung function and maintain it in older adults.^69^ ^98^ Separate golf and swimming interventions decreased hospital admission rates and symptom severity, while improving quality of life and parent satisfaction in a randomised trial of children with asthma.^99^

##### Metabolic health

Quasi-experimental studies are united in describing overall positive effects on lipid profile.^53^ ^57^ ^67^ ^85^ Statistically significant effects of a season of golf on body composition (body weight, body mass index, waist-to-hip ratio and some skinfold thicknesses) are described in controlled trials,^57^ ^85^ while a smaller study showed no effect on body composition.^69^ Blood glucose levels decreased during golfing activity in Swedish and Japanese studies.^53^ ^66^

##### Cancer risk

An inverse relationship is demonstrated regarding physical activity and colon/breast cancer.^4^ Five ultraviolet radiation dosimetry studies report exposures that place golfers at higher risk of skin cancer than non-golfers.^100–104^ A cross-sectional study of female professional and amateur golf players highlighted increased numbers of non-melanoma skin cancers.^105^ Appropriate sunscreen, protective clothing and shade availability are suggested.^100^ ^106^

##### Musculoskeletal health

Golf is associated with musculoskeletal benefits as well as accident and injury. Older golfers may gain improved balance,^70^ ^71^ ^73^ muscular function^72^ and strength^74^ compared to controls, but no lower limb bone mineral density increase was found in male professional golfers.^75^ Female caddies show better bone health than the general female population.^107^ ^108^

#### Golf and injury

Injuries and accidents related to golf comprise the largest group of studies identified by the scoping review. A 2009 systematic review and other reviews describe golf as overall a moderate risk activity for injury compared to other sports.^11^ ^26^

Prospective and retrospective epidemiological studies quote the incidence of injury in amateur golfers annually to be between 15.8% and 40.9%^109–114^ and lifetime injury incidence between 25.2% and 67.4%.^10^ ^115–118^ Prospective longitudinal studies report very low injury rates compared to other sports, at 0.28–0.60 injuries per 1000 hours in amateurs.^57^ ^109^ ^119^ Professionals play more, and are injured more frequently, with annual injury rates of between 31.0% and 90.0%,^115^ ^120^ and quoted lifetime incidence of 60.0–88.5%. Overall, the incidence of injury is moderate, and the rate of injury per hour played is low.

The most frequent cause of injury in amateur and professional golfers is volume of repetitive practice,^113^ ^114^ ^116^ ^117^ ^121^ while suboptimal swing biomechanics are a frequent^115–117^ ^122^ and perhaps even leading^109^ cause in amateurs. Attention to these factors, and to an adequate warm up,^26^ ^123–128^ and physical conditioning^11^ ^26^ ^129^ ^130^ reduces risk of injury.

Regarding limb injuries, the lead side (the left arm and leg in a right-handed golfer) is more often injured than the trail (right side in a right-handed golfer).^11^ ^26^ ^113^ ^131^ ^132^ The mean length of missed practice or competition quoted is 4.0–5.2 weeks.^116^ ^117^ The spine and particularly the lower back account for the greatest overall incidence of injury in amateur golfers (18.3–36.4%).^109^ ^115–117^ ^133^ The elbow (8.0–33.0%), the wrist and hand (10.0–32%) and shoulder (4.0–18.6%) are other frequently injured anatomical regions in amateur golfers.^10^ ^11^ ^109^ ^110^ ^115–117^ ^133^

Golf is an infrequent cause of head and particularly ocular injury, but these injuries can be severe particularly in children.^134–145^ Injuries in children most often occur when struck by a club,^134^ ^140–142^ while adults are more frequently hit by a ball. Most paediatric golf-related injuries occur away from a golf course^134^ ^143^ with authors urging preventative strategies targeting improved education and supervision of children and safe storage of golf equipment.^11^ ^134^ ^135^ ^138^ ^140^

Although still infrequent, golf is reported to be the sport with the highest incidence of lightning strike in the USA^146^ with deaths,^147^ ^148^ and prevention strategies for players and courses outlined.^147^

Golf cart-related injuries, including from falls, collisions or limb entrapment, can occur^11^ ^149^ ^150^ and can be severe.^139^ ^149–152^ The US National Safety Council reports over 15 000 golf cart-related injuries per year, noting that not all are related to golf.^149^ Authors suggest regulation and instruction around safe golf cart use,^150^ ^151^ as well as improvement and standardisation of safety features—for example, speed limiters, seat belts and front wheel brakes.^149^ ^150^

#### Golf and mental health/wellness

No consistent evidence for the associations or effects of golf on mental illness was reported. Golf is associated with positive impacts on mental wellness.^35^ ^153^ ^154^ A wide range of methodologies, including qualitative interviewing, cross-sectional surveys and longitudinal studies, were used.

##### Mental health

A small experimental study enrolling nine persons with severe and enduring mental illness tentatively reported a number of mental and social benefits for participants.^155^ There is conflicting evidence relating to the effect of golf and other sports on mood and anxiety, with positive^156^ and negative^62^ mood changes noted. Improvement in stress and anxiety was reported by two studies^156^ ^157^ highlighting stress-busting qualities, verbalised as a ‘sense of cool control’ and a ‘release of aggression’.^157^ Conversely, studies describe anxieties relating to performance on the golf course.^62^ ^157^ Increased heart rates are noted prior to tournament play, consistent with prematch tension.^86^

##### Mental wellness

Quantitative and qualitative studies have described benefits related to self and group identity^157–161^ and social connections, many of which have been cultured long term.^84^ ^161^ ^162^ Golf facilitated opportunities for intergenerational interaction,^163^ ^164^ and created opportunities to rebuild social connections^86^ ^161^ and confidence^165^ during and post illness.

Self-efficacy, self-worth and physical activity levels improved after a golf intervention in 814 participants with a disability in the USA.^166^ In addition, self-worth in golfing populations^158^ ^161^ and self-esteem^156^ ^167^ in sporting populations that include golfers show positive change. An initial analysis of ‘The First Tee’—an at-scale US sport-based development programme—suggests that participants and parents noted improved confidence, interpersonal skills and emotional control.^168^ Finally, sunshine, fresh air and kinaesthetic pleasure were identified through qualitative interview responses as contributing factors to potential wellness^162^ benefits related to golf.

In summary, a number of qualitative and quantitative studies describe improved wellness in golfers, but there are few controlled studies looking at golf and mental health.

### Further research priorities

This study has identified research gaps in the existing literature on golf and health with future research priorities outlined in table 2.

**Table 2:** Research priorities related to golf and health

Research priority relating to golf	Comment	Why required
Mental health and illness	Physical activity has an overall positive impact on wellness and mental ill health, but robust, controlled studies with objective measures are required in relation to golf	Weight of evidence low
Systematic reviews relating to golf and health	To explore cause and effect nature of the relationships described	Scoping review methods cannot answer these specific questions, but have been able to map the evidence landscape and indicate where more focused study is required
Muscle strengthening/strength and balance/musculoskeletal benefits	Research on the contribution of golf to muscle strengthening/strength and balance, and potential effects in relation to osteoporosis and osteoarthritis could be important to golfers, practitioners and policymakers looking to provide advice to patients and populations	Weight of evidence low/knowledge gap
Golf carts	Research is needed exploring how health effects/relationships differ between golf played while riding a golf cart and golf played walking the course	Weight of evidence low
Spectating	Research assessing useful physical activity accrued spectating is required. Opportunities exist to shape health behaviours among spectators on course and in daily life using the experience as a ‘teachable moment’	Knowledge gap
Health behaviour change	Research is needed addressing how golfers and potential golfers can be influenced to take part and maintain golfing activity, and investigating and improving knowledge and behaviours related to golf injuries, illnesses and accidents	Weight of evidence low
Economic effects	Research investigating cost savings to health and other services associated with golf, and opportunities to make golf more accessible and affordable for all will inform policy	Weight of evidence low
Specific populations	Research addressing associations between golf and health in (1) disabled and (2) older adult populations may highlight specific benefits/disbenefits	Weight of evidence low

### Limitations

Scoping reviews are comprehensive, but not exhaustive in identifying literature^16^ recognising the balance between the breadth and depth of analysis.^169^ Our search was subject to older but relevant sources being less available via databases, search platforms and search engines. Scoping reviews are broad in nature and provide an overview of existing literature regardless of quality, providing a broader and more contextual overview than systematic reviews. Formal assessment of methodological quality is not undertaken when conducting a scoping review,^14^ ^15^ ^169^ and synthesis of the literature quantitatively, nor demonstration of a cause and effect nature for the found relationships is not possible. Golfers are likely different to non-golfers in many ways, with confounding factors a challenge to identify and adequately control. Documented attempts were made throughout the design and conduct of this study to appraise and report evidence in an objective way.^17^ Rigorous and reproducible methods have been applied and authors are committed to publish all findings whether findings were positive, negative or not significant.

## Conclusions

This scoping review identified over 300 studies investigating the relationship between golf and health. Golf has been shown to provide moderate intensity aerobic physical activity and therefore could be expected to have the same beneficial effects on longevity, physical health, mental health and wellness associated with physical activity.^170^ The scoping review cannot demonstrate causative effects, but reports evidence that is biologically plausible and relatively consistent, highlighting positive associations between golf and physical health, and mental wellness. The best available evidence suggests that golf may contribute to reduced mortality. The existing evidence supports efforts to promote golf as a sport with overall health benefits. To maximise health benefits, golfers should walk the course rather than riding a golf cart.

Research assessing golf's contribution to muscle strengthening recommendations, the relationships of golf on mental health, golf spectating and health, and the influencing of health behaviours in golfers, have been identified as priorities for further study. Systematic reviews to further explore health effects of golf on specific conditions are also required.

**What is known?**

- Scoping reviews provide a useful framework to collate and summarise information on a broad topic.
- Golf is played by over 50 million people of all ages worldwide.

**What this study adds?**

- Playing golf can provide moderate intensity physical activity and has overall positive associations with physical health and mental wellness, while golf may contribute to increased longevity.
- Disbenefits include (mostly overuse) injuries; accidents are rare, but deleterious consequences of them can be high.
- Priority areas for future research include the associations and effects of golf on mental health, golf's contribution to muscle strengthening, balance and falls prevention, and influencing health behaviours among golfers and potential golfers. Systematic reviews to further explore the cause and effect nature of the relationships described are merited.

## References

[1] Farrally MR, Cochran AJ, Crews DJ, et al Golf science research at the beginning of the twenty-first century. J Sports Sci 2003;21:753–65. 10.1080/0264041031000102123 14579870

[2] The Royal and Ancient. Golf around the world. The Royal and Ancient, 2015.

[3] World Health Organisation. Health impact assessment—the determinants of health. World Health Organisation, 2011.

[4] Lee IM, Shiroma EJ, Lobelo F, et al Effect of physical inactivity on major non-communicable diseases worldwide: an analysis of burden of disease and life expectancy. Lancet 2012;380:219–29. 10.1016/S0140-6736(12)61031-9 22818936 PMC3645500

[5] Lim SS, Vos T, Flaxman AD, et al A comparative risk assessment of burden of disease and injury attributable to 67 risk factors and risk factor clusters in 21 regions, 1990–2010: a systematic analysis for the Global Burden of Disease Study 2010. Lancet 2012;380:2224–60. 10.1016/S0140-6736(12)61766-8 23245609 PMC4156511

[6] Kohl HW III, Craig CL, Lambert EV, et al The pandemic of physical inactivity: global action for public health. Lancet 2012;380:294–305. 10.1016/S0140-6736(12)60898-8 22818941

[7] National Centre for Social Research. Health survey for England. Health and Social Care Information Centre, 2012.

[8] The Scottish Government. The Scottish Health Survey 2013. The Scottish Government, 2014.

[9] McHardy A, Pollard H, Luo K Golf injuries: a review of the literature. Sports Med 2006;36:171–87. 10.2165/00007256-200636020-00006 16464124

[10] Batt ME Golfing injuries. An overview. Sports Med 1993;16:64–71. 10.2165/00007256-199316010-00006 8356378

[11] Cabri J, Sousa JP, Kots M, et al Golf-related injuries: a systematic review. Eur J Sport Sci 2009;9:353–66. 10.1080/17461390903009141

[12] Walker Research Group. World golf foundation and golf 20/20 commission report on golf's health benefits. Walker Research Group, 2011.

[13] Oja P, Titze S, Kokko S, et al Health benefits of different sport disciplines for adults: systematic review of observational and intervention studies with meta-analysis. Br J Sports Med 2015;49:434–40. 10.1136/bjsports-2014-093885 25568330

[14] Arksey H, O'Malley L Scoping studies: towards a methodological framework. Int J Soc Res Methodol 2005;8:19–32. 10.1080/1364557032000119616

[15] Peters M, Godfrey C, McInerney P, et al The Joanna Briggs Institute Reviewers' Manual 2015: Methodology for JBI Scoping Reviews. The Joanna Briggs Institute, 2015.

[16] Levac D, Colquhoun H, O'Brien KK Scoping studies: advancing the methodology. Implement Sci 2010;5:69 10.1186/1748-5908-5-69 20854677 PMC2954944

[17] Murray A, Daines L, Archibald D, et al The relationship and effects of golf on physical and mental health: a scoping review protocol. Br J Sports Med 2016;50:647–50. 10.1136/bjsports-2015-095914 27130924

[18] Broman G, Thomas P Golf: Exercise for health and longevity. In Thomas PR, Ed. Optimising performance in golf. Australian Academic Press, 2001:149–63.

[19] Sabo D, Snyder M Sports and fitness in the lives of working women golfers: an exploratory study. A special report prepared for the ladies professional golf association. A Special Summary Report Golf Summit, 1991.

[20] Shade CX Teeing up for a healthy heart. Diabetes Self Manag 2011;28:28, 30-2. 21510249

[21] Global Observatory for Physical Activity. Country cards. Global Observatory for Physical Activity, 2016 (accessed Aug 2016).

[22] Giulianotti R, Robertson R The globalization of football: a study in the glocalization of the ‘serious life’. Br J Sociol 2004;55:545–68. 10.1111/j.1468-4446.2004.00037.x 15663424

[23] Pluim BM, Miller S, Dines D, et al Sport science and medicine in tennis. Br J Sports Med 2007;41:703–4. 10.1136/bjsm.2007.040865 17957002 PMC2465261

[24] World Rugby. World Rugby participation statistics. World Rugby, 2016 (accessed Aug 2016).

[25] Petrick J, Backman S, Bixler R, et al Analysis of golfer motivations and constraints by experience use history. J Leisure Res 2001;33:56–70.

[26] Thériault G, Lachance P Golf injuries. An overview. Sports Med 1998;26:43–57. 10.2165/00007256-199826010-00004 9739540

[27] Kolt GS, Driver RP, Giles LC Why older Australians participate in exercise and sport. J Aging Phys Act 2004;12:185–98. 10.1123/japa.12.2.185 15223886

[28] Hulteen RM, Lander NJ, Morgan PJ, et al Validity and reliability of field-based measures for assessing movement skill competency in lifelong physical activities: a systematic review. Sports Med 2015;45:1443–54. 10.1007/s40279-015-0357-0 26173900

[29] Hunt K, Ford G, Mutrie N Is sport for all? Exercise and physical activity patterns in early and late middle age in the West of Scotland. Health Educ 2001;101:151–8.

[30] KPMG Golf Advisory Practice. Golf participation in Europe 2015. KPMG report, 2015.

[31] The Scottish Government. The Scottish Health Survey 2014. The Scottish Government, 2015.

[32] Hallal PC, Andersen LB, Bull FC, et al Global physical activity levels: surveillance progress, pitfalls, and prospects. Lancet 2012;380:247–57. 10.1016/S0140-6736(12)60646-1 22818937

[33] World Health Organisation. Global recommendations on physical activity for health. World Health Organisation, 2010. 26180873

[34] Four Home Countries’ Chief Medical Officers. Start active, stay active. A report on physical activity for health from the four home countries’ chief medical officers. Department of Health, 2011.

[35] US Department of Health and Human Services. 2008 physical activity guidelines for Americans. US Department of Health and Human Services, 2008.

[36] Tangen JO, Sunde A, Sageie J, et al In accordance with governmental recommendations—a study of golf and health. J Sports Sci 2013;1:15–25.

[37] O'Halloran P Exercise prescription in health and disease. Faculty of Sports and Exercise Medicine, 2012.

[38] Ainsworth BE, Herrmann SD, Meckes N, et al 2011 compendium of physical activities: a second update of codes and MET values. Med Sci Sports Exerc 2011;43:1575–81. 10.1249/MSS.0b013e31821ece12 21681120

[39] Moy K, Scragg R, McLean G, et al Metabolic equivalent (MET) intensities of culturally-specific physical activities performed by New Zealanders. N Z Med J 2006;119:U2000. 16751824

[40] Hendelman D, Miller K, Baggett C, et al Validity of accelerometry for the assessment of moderate intensity physical activity in the field. Med Sci Sports Exerc 2000;32(9 Suppl):S442–9. 10.1097/00005768-200009001-00002 10993413

[41] Passmore R, Durnin JV Human energy expenditure. Physiol Rev 1955;35:801–40. 13266530 10.1152/physrev.1955.35.4.801

[42] Lampley JH, Lampley PM, Howley ET Caloric cost of playing golf. Res Q 1977;48:637–9. 270198

[43] Dobrosielski DA, Brubaker PH, Berry MJ, et al The metabolic demand of golf in patients with heart disease and in healthy adults. J Cardiopulm Rehabil 2002;22:96–104. 10.1097/00008483-200203000-00008 11984207

[44] Bassett DR Jr, Ainsworth BE, Swartz AM, et al Validity of four motion sensors in measuring moderate intensity physical activity. Med Sci Sports Exerc 2000;32(9 Suppl):S471–80. 10.1097/00005768-200009001-00006 10993417

[45] Ikeda ER, Cooper L, Gulick P, et al The metabolic cost of carrying a single-versus double-strap golf bag. J Strength Cond Res 2008;22:974–7. 10.1519/JSC.0b013e31816f6f2e 18438213

[46] Taylor HL, Jacobs DR, Schucker B, et al A questionnaire for the assessment of leisure time physical activities. J Chronic Dis 1978;31:741–55. 10.1016/0021-9681(78)90058-9 748370

[47] Zunzer SC, von Duvillard SP, Tschakert G, et al Energy expenditure and sex differences of golf playing. J Sports Sci 2013;31:1045–53. 10.1080/02640414.2013.764465 23362842

[48] Dear JB, Porter MM, Ready AE Energy expenditure during golfing and lawn mowing in older adult men. J Aging Phys Act 2010;18:185–200. 10.1123/japa.18.2.185 20440030

[49] Gabellieri JM The physiological demands of walking during golf [M.S.]. Ann Arbor: University of Rhode Island, 2011.

[50] Haisman MF, Winsmann FR, Goldman RF Energy cost of pushing loaded handcarts. J Appl Physiol 1972;33:181–3. 5054421 10.1152/jappl.1972.33.2.181

[51] Crowell B Energy cost of participation in golf as determined by telemetry. ProQuest Dissertations Publishing, 1970.

[52] McGill S A determination of the energy cost of golf during play. ProQuest Dissertations Publishing, 1963.

[53] Murase Y, Kamei S, Hoshikawa T Heart rate and metabolic responses to participation in golf. J Sports Med Phys Fitness 1989;29:269–72. 2635259

[54] Loy SF The effect of the game of golf on cardiopulmonary fitness of middle-aged men. Northridge: California State University, 1979.

[55] Burkett LN, von Heijne-Fisher U Heart rate and calorie expenditure of golfers carrying their clubs and walking flat and hilly golf courses. Int Sports J 1998;2:78–85.

[56] Getchell LH Energy cost of playing golf. Arch Phys Med Rehabil 1968;49:31–5. 5635201

[57] Parkkari J, Natri A, Kannus P, et al A controlled trial of the health benefits of regular walking on a golf course. Am J Med 2000;109:102–8. 10967150 10.1016/s0002-9343(00)00455-1

[58] Ludlum D, Henry S, Iwaskewcz M, et al Energy expenditure and cardiovascular responses to golf: walking vs riding. *American College of Sports Medicine Conference—abstract*, 2014.

[59] Lyerly G, Epton H, Fitzsimmons K, et al Golf: the effect of walking versus riding on energy expenditure. *American College of Sports Medicine South East Conference—abstract*, 2011.

[60] Kobriger SL, Smith J, Hollman JH, et al The contribution of golf to daily physical activity recommendations: how many steps does it take to complete a round of golf? Mayo Clin Proc 2006;81:1041–3. 10.4065/81.8.1041 16901027

[61] Sanders CM, Broker JP, Berning JR, et al The relationship between golf and walking benefits: a pedometer-based exercise assessment. Med Sci Sports Exerc 2007;39:S384 10.1249/01.mss.0000274510.86518.f5

[62] Lane AM, Jarrett H Mood changes following golf among senior recreational players. J Sports Sci Med 2005;4:47–51. 24431960 PMC3880083

[63] Dear JB Determining energy expenditure during golf and lawn-mowing in older adult males: sufficient for health? Proquest Dissertation Publishing, 2005.

[64] Lyerly GW, Meyler T, Fitzsimmons K, et al Golf: the effect of walking versus utilizing a pull-cart on cardiovascular responses. *American College of Sports Medicine South East Conference—abstract*, 2011.

[65] Fitzsimmons K, Lyerly GW, Beam S, et al Acute cardiovascular responses to playing golf: walking versus riding. *American College of Sports Medicine South East Conference—abstract*, 2014.

[66] Broman G, Johnsson L, Kaijser L Golf: a high intensity interval activity for elderly men. Aging Clin Exp Res 2004;16:375–81. 10.1007/BF03324567 15636463

[67] Stauch M, Liu Y, Giesler M, et al Physical activity level during a round of golf on a hilly course. J Sports Med Phys Fitness 1999;39:321–7. 10726433

[68] Kras J, Larsen B A comparison of the health benefits of walking and riding during a round of golf. Int Sports J 2002;6:112–16.

[69] Getchell L An analysis of the effects of a season of golf on selected cardiovascular, metabolic, and muscular fitness measures on middle-aged men and the caloric cost of golf. ProQuest Dissertations Publishing, 1965.

[70] Tsang WW, Hui-Chan CW Static and dynamic balance control in older golfers. J Aging Phys Act 2010;18:1–13. 10.1123/japa.18.1.1 20181990

[71] Tsang WW, Hui-Chan CW Effects of exercise on joint sense and balance in elderly men: Tai Chi versus golf. Med Sci Sports Exerc 2004;36:658–67. 10.1249/01.MSS.0000122077.87090.2E 15064594

[72] Martínez-Bustelo S, Brown S, Warner M, et al Between-side symmetry of quadriceps thickness using ultrasound imaging in female golfers and non-golfers aged over 80 years. *Conference proceeding, Osteoarthritis Research Society International 2016 World Congress—abstract*, 2016.

[73] Gao KL, Hui-Chan CW, Tsang WW Golfers have better balance control and confidence than healthy controls. Eur J Appl Physiol 2011;111:2805–12. 10.1007/s00421-011-1910-7 21416145

[74] Sell TC, Tsai YS, Smoliga JM, et al Strength, flexibility, and balance characteristics of highly proficient golfers. J Strength Cond Res 2007;21:1166–71. 18076270 10.1519/R-21826.1

[75] Dorado C, Sanchis Moysi J, Vicente G, et al Bone mass, bone mineral density and muscle mass in professional golfers. J Sports Sci 2002;20:591–7. 10.1080/026404102320183149 12190278

[76] Sedentary Behaviour Research Network. Standardized use of the terms “sedentary” and “sedentary behaviours”. Appl Physiol Nutr Metab 2012;37:540–2. 22540258 10.1139/h2012-024

[77] Hansen H, Gauthier R The professional golf product: spectators’ views. Sport Market Q 1994;3:9–16.

[78] Hansen H, Gauthier R Spectators’ views of LPGA golf events. Sport Market Q 1993;2:17–25.

[79] Lyu SO, Lee H Market segmentation of golf event spectators using leisure benefits. J Trav Tourism Market 2013;30:186–200. 10.1080/10548408.2013.774913

[80] Farahmand B, Broman G, de Faire U, et al Golf: a game of life and death—reduced mortality in Swedish golf players. Scand J Med Sci Sports 2009;19:419–24. 10.1111/j.1600-0838.2008.00814.x 18510595

[81] Lee IM, Sesso HD, Oguma Y, et al The “weekend warrior” and risk of mortality. Am J Epidemiol 2004;160:636–41. 10.1093/aje/kwh274 15383407

[82] Coate D, Schwenkenberg J Survival function estimates for champions tour golfers. J Sports Econ 2013;14:656–63. 10.1177/1527002512451583

[83] Wen CP, Wai JP, Tsai MK, et al Minimum amount of physical activity for reduced mortality and extended life expectancy: a prospective cohort study. Lancet 2011;378:1244–53. 10.1016/S0140-6736(11)60749-6 21846575

[84] Yang PF A comparison of self-reported health conditions and exercise habits among middle aged male golfers in southern Alabama [D.S.M.]. Ann Arbor: United States Sports Academy, 2008.

[85] Palank EA, Hargreaves EH Jr The benefits of walking the golf course. Phys Sportsmed 1990;18:77–80. 10.1080/00913847.1990.11710155 27452057

[86] Unverdorben M, Kolb M, Bauer I, et al Cardiovascular load of competitive golf in cardiac patients and healthy controls. Med Sci Sports Exerc 2000;32:1674–8. 10.1097/00005768-200010000-00002 11039636

[87] Unverdorben M, Bauer U, Bauer I, et al Golf in the rehabilitation of cardiac patients. Herz Kreislauf 1998;30:99–102.

[88] The Editor. Therapeutic golf puts patients back in the swing. Hosp Peer Rev 1995;20:58–60. 10153192

[89] Lemaitre RN, Siscovick DS, Raghunathan TE, et al Leisure-time physical activity and the risk of primary cardiac arrest. Arch Intern Med 1999;159:686 10.1001/archinte.159.7.686 10218747

[90] Fujiwara M, Asakuma S, Nakamura K, et al [Acute myocardial infarction during sport]. J Cardiol 1995;26:213–17. 7500263

[91] Quigley F A survey of the causes of sudden death in sport in the Republic of Ireland. Br J Sports Med 2000;34:258–61. 10.1136/bjsm.34.4.258 10953896 PMC1724206

[92] Ragosta M, Crabtree J, Sturner WQ, et al Death during recreational exercise in the State of Rhode Island. Med Sci Sports Exerc 1984;16:339–42. 10.1249/00005768-198408000-00003 6493012

[93] Reed DB, Birnbaum A, Brown LH, et al Location of cardiac arrests in the public access defibrillation trial. Prehosp Emerg Care 2006;10:61–76. 10.1080/10903120500366128 16526143

[94] Eckstein M The Los Angeles public access defibrillator (PAD) program: ten years after. Resuscitation 2012;83:1411–12. 10.1016/j.resuscitation.2012.03.029 22484436

[95] Lucas J, Davila AA, Waninger KN, et al Cardiac arrest on the links: are we up to par? Availability of automated external defibrillators on golf courses in southeastern Pennsylvania. Prehosp Disaster Med 2006;21:112–14. 16771002 10.1017/s1049023x00003459

[96] Muraoka H, Ohishi Y, Hazui H, et al Location of out-of-hospital cardiac arrests in Takatsuki City: where should automated external defibrillator be placed. Circ J 2006;70:827–31. 10.1253/circj.70.827 16799233

[97] Choi MH, Hong JM, Lee JS, et al Preferential location for arterial dissection presenting as golf-related stroke. AJNR Am J Neuroradiol 2014;35:323–6. 10.3174/ajnr.A3768 24184518 PMC7965742

[98] Brown S, Samuel D, Agyapong-Badu S, et al Age related differences in lung function between female recreational golfers and less active. *Proceedings of the World Scientific Congress on Golf*, 2016 (in press).

[99] Weisgerber M, Webber K, Meurer J, et al Moderate and vigorous exercise programs in children with asthma: safety, parental satisfaction, and asthma outcomes. Pediatr Pulmonol 2008;43:1175–82. 10.1002/ppul.20895 19003892

[100] Downs N, Parisi A, Schouten P Basal and squamous cell carcinoma risks for golfers: an assessment of the influence of tee time for latitudes in the Northern and Southern hemispheres. J Photochem Photobiol B 2011;105:98–105. 10.1016/j.jphotobiol.2011.07.007 21862342

[101] Downs NJ, Schouten PW, Parisi AV, et al Measurements of the upper body ultraviolet exposure to golfers: non-melanoma skin cancer risk, and the potential benefits of exposure to sunlight. Photodermatol Photoimmunol Photomed 2009;25:317–24. 10.1111/j.1600-0781.2009.00472.x 19906167

[102] Gurrea Ysasi G, Moreno JC, Serrano MA Ultraviolet erythematic radiation dose received by golfers in winter, in Valencia. Photochem Photobiol 2014;90:1170–3. 10.1111/php.12295 24893676

[103] Sung H, Slocum AC UV radiation exposure to body sites of golfers and effects of clothing. Fam Consum Sci Res J 2006;34:386–400. 10.1177/1077727X06286416

[104] Sung H Golfers’ UV exposure, health beliefs and practices, and intention to adopt UV protective clothing. Ann Arbor: Michigan State University, 2003.

[105] Hanke CW, Zollinger TW, O'Brian JJ, et al Skin cancer in professional and amateur female golfers. Phys Sportsmed 1985;13:51–68. 10.1080/00913847.1985.11708857 27442737

[106] Shuliak-Wills L, Navarro K A community intervention plan to prevent skin cancer in male golfers. Can Oncol Nurs J 2000;10:109–11. 11894278

[107] Goto S, Ishima M, Shimizu M, et al A longitudinal study for femoral neck bone mineral density increases in premenopausal caddies using dual-energy X-ray absorptiometry. J Bone Miner Metab 2001;19:125–30. 10.1007/s007740170051 11281161

[108] Hoshino H, Kushida K, Yamazaki K, et al Effect of physical activity as a caddie on ultrasound measurements of the Os calcis: a cross-sectional comparison. J Bone Miner Res 1996;11:412–18. 10.1002/jbmr.5650110316 8852953

[109] McHardy A, Pollard H, Luo K One-year follow-up study on golf injuries in Australian amateur golfers. Am J Sports Med 2007;35:1354–60. 10.1177/0363546507300188 17387218

[110] McHardy A, Pollard H The epidemiology of golf-related injuries in Australian amateur golfers. Med Sci Sports Exerc 2006;38:S350 10.1249/00005768-200605001-01491 17387218

[111] Lee H-J, Yong-Seok J Golf and injury incidence in recreational golfers: a retrospective study. J Convergence Inf Technol 2013;8:522.

[112] Eisenhart C, Fradkin A To practice or to play: is golf participation associated with an increased risk of injury? Med Sci Sports Exerc 2011;43:357 10.1249/MSS.0b013e3181ed61a3 20581716 PMC3184184

[113] Fradkin AJ, Windley TC, Myers JB, et al Describing the epidemiology and associated age, gender and handicap comparisons of golfing injuries. Int J Inj Contr Saf Promot 2007;14:264–6. 10.1080/17457300701722585 18075877

[114] Fradkin AJ, Cameron PA, Gabbe BJ Golf injuries—common and potentially avoidable. J Sci Med Sport 2005;8:163–70. 10.1016/S1440-2440(05)80007-6 16075776

[115] Theriault G, Lacoste E, Gadoury M, et al Golf injury characteristics: a survey from 528 golfers. Med Sci Sports Exerc 1996;28:65 10.1097/00005768-199605001-00389

[116] Gosheger G, Liem D, Ludwig K, et al Injuries and overuse syndromes in golf. Am J Sports Med 2003;31:438–43. 12750140 10.1177/03635465030310031901

[117] McCarroll JR, Rettig AC, Shelbourne KD Injuries in the amateur golfer. Phys Sportsmed 1990;18:122–6. 10.1080/00913847.1990.11709999 27464054

[118] Nicholas JJ, Reidy M, Oleske DM An epidemiologic survey of injury in golfers. J Sport Rehabil 1998;7:112–21. 10.1123/jsr.7.2.112

[119] Parkkari J, Kannus P, Natri A, et al Active living and injury risk. Int J Sports Med 2004;25:209–16. 10.1055/s-2004-819935 15088246

[120] Barclay C, West S, Shoaib Q, et al Injuries patterns among professional golfers: an international survey. Br J Sports Med 2011;45:e1 10.1136/bjsm.2010.081554.12

[121] McCarroll JR, Gioe TJ Professional golfers and the price they pay. Phys Sportsmed 1982;10:64–8. 29267100 10.1080/00913847.1982.11947272

[122] McCarroll JR The frequency of golf injuries. Clin Sports Med 1996;15:1–7. 8903705

[123] Fradkin AJ, Windley TC, Myers JB, et al, eds Describing the warm-up habits of recreational golfers and the associated injury risk. Science and Golf V Proceedings of the Fifth World Scientific Congress of Golf. Mesa (AZ): Energy in Motion, 2008.

[124] Fradkin AJ, Finch CF, Sherman CA Warm-up attitudes and behaviours of amateur golfers. J Sci Med Sport 2003;6:210–15. 10.1016/S1440-2440(03)80256-6 12945627

[125] Fradkin AJ, Finch CF, Sherman CA Warm up practices of golfers: are they adequate? Br J Sports Med 2001;35:125 10.1136/bjsm.35.2.125 11273975 PMC1724309

[126] Versteegh TH, Vandervoort AA, Lindsay DM, et al Fitness, performance and injury prevention strategies for the senior golfer. Int J Sports Sci Coach 2008;3:199–214. 10.1260/174795408785024162

[127] Dhillon M, Singh S, Dhillon H, et al Epidemiology of golf related musculo-skeletal injuries. Indian J Orthop 2006;40:188–90. 10.4103/0019-5413.34491

[128] Smith MF, Hillman R A retrospective service audit of a mobile physiotherapy unit on the PGA European Golf Tour. Phys Ther Sport 2012;13:41–4. 10.1016/j.ptsp.2010.09.001 22261430

[129] Sherman CA, Finch CF Preventing injuries to competitive and recreational adult golfers: what is the evidence? J Sci Med Sport 2000;3:65–78. 10.1016/S1440-2440(00)80049-3 10839230

[130] Meira EP, Brumitt J Minimizing injuries and enhancing performance in golf through training programs. Sports Health 2010;2:337 10.1177/1941738110365129 23015957 PMC3445090

[131] Jacobson JA, Miller BS, Morag Y Golf and racquet sports injuries. Semin Musculoskelet Radiol 2005;9:346–59. 10.1055/s-2005-923379 16315117

[132] Hawkes R, O'Connor P, Campbell D The prevalence, variety and impact of wrist problems in elite professional golfers on the European Tour. Br J Sports Med 2013;47:1075–9. 10.1136/bjsports-2012-091917 24014125 PMC3812892

[133] Batt ME A survey of golf injuries in amateur golfers. Br J Sports Med 1992;26:63–5. 10.1136/bjsm.26.1.63 1600459 PMC1478983

[134] Fountas KN, Kapsalaki EZ, Machinis TG, et al Pediatric golf-related head injuries. Childs Nerv Syst 2006;22:1282–7. 10.1007/s00381-006-0077-8 16598498

[135] Fradkin AJ, Cameron PA, Gabbe BJ Children's misadventures with golfing equipment. Int J Inj Contr Saf Promot 2005;12:201–3. 10.1080/17457300500088972 16335440

[136] Brian R, Glazer G Taming the little tigers. Golf-related head injuries in children. Adv Nurse Pract 2005;13:59–60, 62. 15984361

[137] Finch C, Sherman C, James T The epidemiology of golf injuries in Victoria, Australia: evidence from sports medicine clinics and emergency department presentations. Science and golf III: proceedings of the 1998 World Scientific Congress of Golf Human Kinetics, 1999:73–82.

[138] Vitale MA, Mertz KJ, Gaines B, et al Morbidity associated with golf-related injuries among children: findings from a pediatric trauma center. Pediatr Emerg Care 2011;27:11–12. 10.1097/PEC.0b013e3182037c9a 21206251

[139] Rahimi SY, Singh H, Yeh DJ, et al Golf-associated head injury in the pediatric population: a common sports injury. J Neurosurg 2005;102(2 Suppl):163–6. 10.3171/jns.2005.102.2.0163 16156225

[140] McGuffie AC, Fitzpatrick MO, Hall D Golf related head injuries in children: the little tigers. Scott Med J 1998;43:139–40. 9854298 10.1177/003693309804300504

[141] Brennan PO Golf related head-injuries in children. BMJ 1991;303:54 10.1136/bmj.303.6793.54-b PMC1670280 1859965

[142] Lindsay KW, McLatchie G, Jennett B Serious head injury in sport. Br Med J 1980;281:789–91. 10.1136/bmj.281.6243.789 7427450 PMC1714033

[143] Wang A, Cohen AR, Robinson S The “swing-ding”: a golf-related head injury in children. J Neurosurg Pediatr 2011;7:111–15. 10.3171/2010.10.PEDS10283 21194295

[144] Delilbasi C, Yamazawa M, Nomura K, et al Maxillofacial fractures sustained during sports played with a ball. Oral Surg Oral Med Oral Pathol Oral Radiol Endod 2004;97:23–7. 10.1016/j.tripleo.2003.10.008 14716253

[145] Rosenow JM, Hahn MS, Moore KD, et al Pediatric cranial golf injuries—an emerging contemporary phenomenon? Surg Neurol 1998;50:608. 9870826

[146] Cherington M, Vervalin C Lightning injuries—who is at greatest risk? Phys Sportsmed 1990;18:58–61. 10.1080/00913847.1990.11710113 27447386

[147] Cherington M Lightning injuries in sports: situations to avoid. Sports Med 2001;31:301–8. 10.2165/00007256-200131040-00004 11310549

[148] Zack F, Raphael T, Kupfer J, et al Four fatalities due to lightning on a golf course. Rechtsmedizin 2013;23:114–18. 10.1007/s00194-013-0870-0

[149] Watson DS, Mehan TJ, Smith GA, et al Golf cart-related injuries in the U.S. Am J Prev Med 2008;35:55–9. 10.1016/j.amepre.2008.03.029 18541177

[150] McGwin G Jr, Zoghby JT, Griffin R, et al Incidence of golf cart-related injury in the United States. J Trauma 2008;64:1562–6. 10.1097/TA.0b013e3181238d39 18545124

[151] Tung MY, Hong A, Chan C Golf buggy related head injuries. Singapore Med J 2000;41:504–5. 11281444

[152] Miller BL, Waller JL, McKinnon BJ Craniofacial injuries due to golf cart trauma. Otolaryngol Head Neck Surg 2011;144:883–7. 10.1177/0194599810396790 21493357

[153] Kruger J, Bowles HR, Jones DA, et al Health-related quality of life, BMI and physical activity among US adults (>/=18 years): National Physical Activity and Weight Loss Survey, 2002. Int J Obes (Lond) 2006;31:321 10.1038/sj.ijo.0803386 16703001

[154] Das P, Horton R Rethinking our approach to physical activity. Lancet 2012;380:189–90. 10.1016/S0140-6736(12)61024-1 22818931

[155] Carless D, Douglas K A golf programme for people with severe and enduring mental health problems. J Publ Ment Health 2004;3:26–39. 10.1108/17465729200400026

[156] Belanger LJ, Plotnikoff RC, Clark AM, et al Prevalence, correlates, and psychosocial outcomes of sport participation in young adult cancer survivors. Psychol Sport Exerc 2013;14:298–304. 10.1016/j.psychsport.2012.10.010

[157] Adatto C On play and the psychopathology of golf. J Am Psychoanal Assoc 1964;12:826–41. 10.1177/000306516401200408 14221040

[158] Paul JF The experience of playing golf: a heuristic psychological study. Diss Abstr Int 1991;51:5586.

[159] Walker HJ An investigation into the personal meaning of golf [Ph.D.]. Ann Arbor: The Ohio State University, 1989.

[160] Austin M Constructing the active-body: a sociological investigation [Ph.D.]. Ann Arbor: Oxford Brookes University (United Kingdom), 2003.

[161] Beard DS Psychological factors impeding older men from returning to recreational golf after knee joint replacement surgery [Ph.D.]. Ann Arbor: Capella University, 2007.

[162] Berlin KL, Klenosky DB Let Me Play, Not Exercise! A laddering study of older women's motivations for continued engagement in sports-based versus exercise-based leisure time physical activities. J Leisure Res 2014;46:127–52.

[163] Cann AP, Vandervoort AA, Lindsay DM Optimizing the benefits versus risks of golf participation by older people. J Geriatr Phys Ther 2005;28:85–92. 10.1519/00139143-200512000-00004 16386170

[164] Kleiber DA Redeeming leisure in later life. Positive leisure science: from subjective experience to social contexts. Springer Science + Business Media, 2013:21–38.

[165] Hoberty RJ, Craig MW “Living up to par”—a golf tournament for persons with COPD. Respir Care 1983;28:1480–3. 10315482

[166] Kim K, Compton DM, Robb GM Increasing the self-efficacy of individuals with a disability through a theory-based curriculum applied to playing golf. Int J Disabil Hum Dev 2011;10:151–7.

[167] Ekeland E, Heian F, Hagen KB, et al Can exercise improve self esteem in children and young people? A systematic review of randomised controlled trials. Br J Sports Med 2005;39:792–8; discussion 792–8. 16244186 10.1136/bjsm.2004.017707 PMC1725055

[168] Weiss MR, Stuntz CP, Bhalla JA, et al ‘More than a game’: impact of The First Tee life skills programme on positive youth development: project introduction and Year 1 findings. Qual Res Sport Exer Health 2013;5:214–44. 10.1080/2159676X.2012.712997

[169] Pham MT, Rajić A, Greig JD, et al A scoping review of scoping reviews: advancing the approach and enhancing the consistency. Res Synth Methods 2014;5: 371–85. 10.1002/jrsm.1123 26052958 PMC4491356

[170] Murray A, Daines L, Archibald D, et al Infographic. Golf and health. Br J Sports Med 2017;51:13–4. 10.1002/jrsm.1123 27697934

