# Supplementary material for: The relationships between golf and health: a scoping review
Source: Br J Sports Med. 2016 Oct 3;51(1):12–9. doi: 10.1136/bjsports-2016-096625 (PMC5256129; doi:10.1136/bjsports-2016-096625)
Supplement: Supplementary appendix [file bjsports-2016-096625supp_appendix1.pdf]

Appendix 1 (online only)- background information on golf

What is golf?

Golf is a sport traditionally played on a large open-air course, in which a ball is struck by a club, with the aim of taking the lowest number of strikes possible to get the ball into (traditionally) 18 holes in the ground. The objective of golf is to get the golf ball into the holes in the lowest number of shots. Most commonly played as a round of 18 holes, golf can also be played over 9 holes, or practiced at driving ranges, putting greens and other golfing facilities. Courses can be of varied topography.

Participation

Golf is played by around 55 million people (1) young and old, on 34,011 golf facilities in 206 of 239 countries worldwide (2). Of these facilities, 71% are open to the public, while 79% of facilities are located in the top 10 golfing countries which are largely based in North America, Europe and Oceania (2). Golf facilities are currently strongest in “developed” nations, with a population of 1210 per golf hole in Oceania, compared with 89,229 per golf hole in Africa (2).

Health Survey for England 2012 (3) data outlines that 2.2% of persons aged 16 and over reported playing golf in the four weeks prior to the survey, although participation levels are greater in higher socio-economic groups. Scottish Health Survey 2013 (4) data highlights that older golfers play more often than younger golfers. Making golf accessible for all is a key challenge for the golf industry.

How golf compares to other sports

Table 1 below highlights the Metabolic Equivalent of Task (MET) for selected popular sports. For all of these activities, exercise intensity can vary considerably during play, and between individuals. The exercise intensity during golf is lowest when standing waiting for others or putting, and higher when swinging a club or walking.

Table 1. Metabolic Equivalent of Task from 2011 Compendium of Physical Activities (5) for selected sports.

Sport	Golf	Tennis (general)	Football (competitive)	Swimming (light or moderate effort)	Cycling (leisure, 9.4 mph)
METs (3)	4.8	7.3	10.0	5.8	5.8

Few reliable statistics are available comparing global participation in sport. Football is recognised as the most popular sport with an estimated 250 million directly participating(6). The most popular five participation sports in England are shown in table 2.

Table 2. Most popular participation sports in England (7), by volume of participants.

Ranking by participation	England
1	Swimming
2	Athletics
3	Cycling
4	Football
5	Golf

Sources:

1. Farrally MR, Cochran AJ, Crews DJ, *et al*. Golf science research at the beginning of the twenty-first century. J Sports Sci 2003;21:753–65.

2. The Royal and Ancient. Golf Around the World. Online. 2015.

3. National Centre Social Research. Health Survey for England 2012. Online- Health and Social Care Information Centre. 2012.

4. The Scottish Government. Scottish Health Survey 2013. The Scottish Government. 2014.

5. Ainsworth BE, Herrmann SD, Meckes N, Greer JL, Vezina J, Haskell WL, et al. 2011 compendium of physical activities: A second update of codes and MET values. Medicine and Science in Sports and Exercise. 2011;43(8):1575-81.

6. Giulianotti RR, R. The globalization of football: a study in the glocalization of the ‘serious life’. Br J Sociology. 2004;55(4):545-68.

7. Sport England. Active People Survey, 10Q2. Sport England. 2016.
