# Supplementary material for: The relationships between golf and health: a scoping review
Source: Br J Sports Med. 2016 Oct 3;51(1):12–9. doi: 10.1136/bjsports-2016-096625 (PMC5256129; doi:10.1136/bjsports-2016-096625)
Supplement: Supplementary appendix [file bjsports-2016-096625supp_appendix3.pdf]

**1. BIBLIOGRAPHIC INFORMATION**	
Study ID	
Article title	
Extracted by	
Type of publication (journal article, book chapter, grey literature)	
Country	
**2. RESEARCHER DETAILS**	
Authors and affiliations (list as presented on paper)	
**3.AIMS & METHODS**	
Study aims	
Methodology	
Methods	
**4.SCOPING REVIEW PCC**	
Population	
Concept	
Context	
**5. A PRIORI THEMES (does the paper report any data relevant to the following issues?)**	
**(a) Golf participation**	
**(b) Physical Activity & Health**	
**(c) Golf and physical activity**	
**(d) Longevity**	
**(e) Cardio-respiratory**	
**(f) Metabolic**	
**(g) MSK**	
**(h) Accident**	
**(i) Illness**	
**(j) Wellness**	
**(k) Mental illness**	
**(l) Special populations**	
**6. EMERGENT THEMES (does the paper report on any further issues not related to the above**	

**that might be of interest to this review?)**	
**(a)**	
**(b)**	
**(c)**	
**(d)**	
